# Efficiency of an artificial intelligence-aided portable 12-lead electrocardiogram acquisition system

**DOI:** 10.1016/j.hroo.2025.05.025

**Published:** 2025-06-02

**Authors:** Meghna Gaddam, Graham Peigh, Shifa Banani, Rod S. Passman

**Affiliations:** Division of Cardiology, Northwestern Medicine, Chicago, Illinois

**Keywords:** 12-lead ECG, Artificial intelligence, Efficiency, Performance, Portable ECG

**Key Findings**

▪ The Kardia 12L (AliveCor, Mountain View, CA) is a US Food and Drug Administration-approved, artificial intelligence-aided, portable electrocardiogram (ECG) acquisition system that produces a 12-lead ECG with application of only 5 surface leads.
▪ In this study, utilization of Kardia 12L significantly reduced ECG acquisition time compared with standard 12-lead ECGs (7.1 minute vs 10.0 minutes, *P* < .001).
▪ Kardia 12L signal quality was sufficient for clinical use in > 90% of patients.

Although a 12-lead (12L) electrocardiogram (ECG) has been the cornerstone diagnostic tool for arrhythmia diagnosis for more than a century, proper acquisition can be time-consuming and intrusive, and is not always universally available owing to device size and cost.^1^^,^^2^ The Kardia 12L (AliveCor, Mountain View, CA) is a US Food and Drug Administration-approved portable, artificial intelligence-aided, ECG system that generates a full 12-lead ECG with only 5 electrodes (Figure 1A and 1B). When placing the leads, the provider is instructed on lead configuration (either primary configuration of V2, V4, left arm, left leg, and right arm or secondary configuration of V1, V4, left arm, left leg, and right arm) (Figure 1B). Primary configuration is recommended for patients presenting with symptoms suggestive of ischemia, or patients without symptoms. Secondary configuration is recommended for patients with a suspicion for arrhythmia. The system records the standard 4 ECG leads from the patient (I, II, and V1 or V2 and V4), derives 4 standard leads (aVL, aVR, aVF, and III) using the standard relationships, and synthesizes 4 remaining leads (V2 or V1, V3, V5, and V6) using a machine learning model to create a 12-lead ECG recording) (Figure 1B). After acquisition, Kardia 12L ECGs are transmitted through Bluetooth to a smart device (phone or tablet) and may be incorporated into standard electronic ECG depository systems.

**Figure 1:**
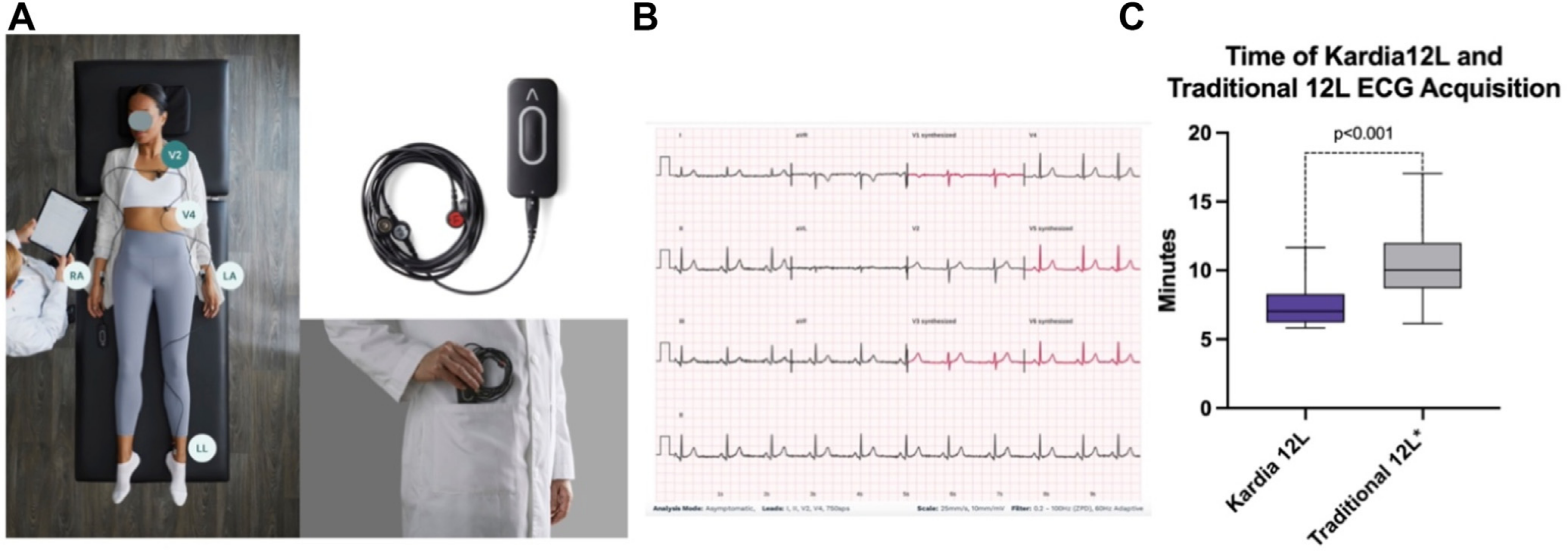
An Artificial Intelligence Aided Portable 12-Lead ECG Acquisition System: Kardia 12L. **A:** Kardia 12L device and primary lead positioning, which does not require the patient to disrobe. **B:** Kardia 12L ECG with direct tracings of the limb leads, V2 and V4, and artificial intelligence interpretation of leads V1, V3, V5, and V6. **C:** Use of Kardia 12L is associated with significantly shorter times of acquisition than those of traditional 12L ECGs. ∗Data from Gaddam and colleagues.^3^ KardiaMobile 6L vs 12-lead ECG: Effects on clinic utilization time. *Journal of Cardiovascular Electrophysiology*. ECG = electrocardiogram.

This study evaluates the efficiency of the Kardia 12L system compared with standard 12L ECGs in the outpatient clinical setting. The secondary objective was to evaluate the performance of the Kardia 12L ECGs, defined as a treating electrophysiologist being satisfied with the quality of the Kardia 12L tracing and not requesting an additional standard 12L ECG.

Patients consecutive in visiting the electrophysiology clinic at Northwestern Memorial Hospital had a Kardia 12L ECG obtained instead of a standard 12-lead ECG. The time of ECG acquisition was measured by a trained observer blinded to the hypothesis of the study and compared with control groups who had only a standard 12L ECG.^3^ Performance was evaluated by assessing the number of patients who necessitated an additional standard 12L ECG at the discretion of the ordering physician. All ECGs were performed by trained clinical staff after a standardized protocol, and data were recorded in an institutional review board-approved database. The research reported in this article adhered to the principles outlined in the Declaration of Helsinki. The study protocol was approved by the Northwestern University institutional review board, and all patients provided written informed consent prior to participation.

In total, 50 patients (67 ± 16.2 years, 58% men) had a Kardia 12L ECG. The primary indication for electrophysiology clinic appointments in this cohort was for evaluation of atrial fibrillation or atrial flutter (74%) or premature ventricular contractions (6%). The most common rhythms recorded on the Kardia 12L ECG were normal sinus rhythm (60%), atrial and/or ventricular paced (20%), atrial fibrillation (10%), and sinus rhythm with premature ventricular contractions (10%).

The average time of Kardia 12L ECG acquisition was significantly shorter than that of a standard 12L ECG (Kardia 12L: 7.1 [6.2–8.3] minutes; standard 12L: 10.0 [8.7–12.0] minutes, *P* < .001; Figure 1C).^3^ There were 5 patients (10%) who initially had a Kardia 12L ECG obtained and necessitated an additional standard 12L ECG thereafter. The reasons for additional ECG acquisition included poor signal quality (n = 2) and inability to definitively measure the QT interval owing to printer settings (n = 3).

To the best of our knowledge, these data are the first to describe the efficiency and performance of the Kardia 12L ECG system in the clinical setting. Results indicate that Kardia 12L reduces the time required for ECG recording compared with a standard 12-lead ECG. These reductions in ECG acquisition time may both increase the amount of time that patients may directly interact with a physician and have implications for clinic efficiency. Indeed, when considering the number of ECGs routinely obtained in clinic, even modest reductions in individual acquisition times may compound over the course of a given day. Importantly, the signal quality achieved by the Kardia 12L ECG system is largely comparable to that of a standard 12L ECG, as shown by the low number of additional 12L ECGs that needed to be obtained. Prior analyses have indicated that there is a very strong correlation between interval measurements and R wave axis determinations produced by Kardia 12L and standard 12L ECGs.^4^

Limitations of this study include the use of highly trained ECG technicians, which may have generated shorter data acquisition times than may be seen in some settings, in addition to unblinding of the trained observed and overreading physicians.

In conclusion, Kardia 12L offers a significant time advantage over traditional 12L ECGs while maintaining diagnostic reliability. The low necessity for follow-up dedicated 12L ECGs supports its role as an efficient alternative in various clinical settings. Taken together, results suggest that use of Kardia 12L may improve workflow efficiency in clinical settings and offers a portable, 12-lead option in environments where standard ECGs are not widely available.

## References

[1] Tai-Seale M., McGuire T.G., Zhang W. (2007). Time allocation in primary care office visits. Health Serv Res.

[2] Lin Y.T., Chen H.A., Wu H.Y., Fan C.M., Hsu J.C., Chen K.C. (2023). Influence of the door-to-ECG time on the prognosis of patients with acute coronary syndrome. Acta Cardiol Sin.

[3] Gaddam M., Liu A., Lohrmann G., Breed A., Passman R. (2024). KardiaMobile 6L versus 12-lead ECG: effects on clinic utilization time. J Cardiovasc Electrophysiol.

[4] Whyte S., Sample K., Mustafina I. (2024). MP-470548-001 diagnostic accuracy of a mobile, artificial intelligence-guided, 12-lead ECG device. Heart Rhythm.

